# N_2_O Emission from Degraded Soybean Nodules Depends on Denitrification by *Bradyrhizobium japonicum* and Other Microbes in the Rhizosphere

**DOI:** 10.1264/jsme2.ME12100

**Published:** 2012-10-05

**Authors:** Shoko Inaba, Fumio Ikenishi, Manabu Itakura, Masakazu Kikuchi, Shima Eda, Naohiko Chiba, Chie Katsuyama, Yuichi Suwa, Hisayuki Mitsui, Kiwamu Minamisawa

**Affiliations:** 1 Graduate School of Life Sciences, Tohoku University, 2–1–1 Katahira, Aoba-ku, Sendai 980–8577, Japan; 2 Department of Biological Sciences, Faculty of Science and Engineering, Chuo University, 1–13–27 Kasuga, Bunkyo-ku, Tokyo 112–8551, Japan

**Keywords:** Nitrous oxide, *Bradyrhizobium japonicum*, Denitrification, *nosZ* gene, Soybean rhizosphere

## Abstract

A model system developed to produce N_2_O emissions from degrading soybean nodules in the laboratory was used to clarify the mechanism of N_2_O emission from soybean fields. Soybean plants inoculated with *nosZ*-defective strains of *Bradyrhizobium japonicum* USDA110 (Δ*nosZ*, lacking N_2_O reductase) were grown in aseptic jars. After 30 days, shoot decapitation (D, to promote nodule degradation), soil addition (S, to supply soil microbes), or both (DS) were applied. N_2_O was emitted only with DS treatment. Thus, both soil microbes and nodule degradation are required for the emission of N_2_O from the soybean rhizosphere. The N_2_O flux peaked 15 days after DS treatment. Nitrate addition markedly enhanced N_2_O emission. A ^15^N tracer experiment indicated that N_2_O was derived from N fixed in the nodules. To evaluate the contribution of bradyrhizobia, N_2_O emission was compared between a *nirK* mutant (Δ*nirK*Δ*nosZ*, lacking nitrite reductase) and Δ*nosZ*. The N_2_O flux from the Δ*nirK*Δ*nosZ* rhizosphere was significantly lower than that from Δ*nosZ*, but was still 40% to 60% of that of Δ*nosZ*, suggesting that N_2_O emission is due to both *B. japonicum* and other soil microorganisms. Only *nosZ*-competent *B. japonicum* (*nosZ*+ strain) could take up N_2_O. Therefore, during nodule degradation, both *B. japonicum* and other soil microorganisms release N_2_O from nodule N via their denitrification processes (N_2_O source), whereas *nosZ*-competent *B. japonicum* exclusively takes up N_2_O (N_2_O sink). Net N_2_O flux from soybean rhizosphere is likely determined by the balance of N_2_O source and sink.

Nitrous oxide (N_2_O) is a key atmospheric greenhouse gas that contributes to global warming and the destruction of stratospheric ozone (14, 46, 47). Agricultural land is a major source of N_2_O through the microbial transformation of nitrogen in the soil (13, 24, 58), and contributes significantly to the net increase in atmospheric N_2_O (46). Legume crops emit more N_2_O than non-legumes (10, 15, 32).

Yang and Cai (55) reported that the emission of N_2_O from a soybean field greatly increased in the late growth period, suggesting that senescence and the decomposition of roots and nodules contributed to emissions. Ciampitti *et al.* (7) also reported marked N_2_O emissions from a soybean field in the late growth period regardless of N fertilization. N_2_O emission from a field with nodulating soybeans was several times higher than that from a field with non-nodulating soybeans (27). N_2_O was emitted directly from degraded nodules of field-grown soybeans in the late growth period (20). Thus, soybean nodules emit N_2_O under field conditions, although the mechanism remains unresolved.

Microorganisms might be involved, as N_2_O can be generated by several microbial processes (4, 13). Using microbial community analysis, Inaba *et al.* (20) nominated potential N_2_O producers that increased in abundance in degraded nodules. Among them, *Bradyrhizobium japonicum* was one of the dominant microbes as endosymbionts of soybean nodules and rhizosphere soil bacteria (9, 29, 30, 33, 35, 39). It reduces nitrogen oxides during denitrification as

$${{\text{NO}}_{3}}^{-}\to {{\text{NO}}_{2}}^{-}\to \text{NO}\to {\text{N}}_{2}\text{O}\to {\text{N}}_{2},$$

where each step is catalyzed by specific reductases. These reductases are encoded, respectively, by *napA* (encoding periplasmic nitrate reductase), *nirK* (Cu-containing nitrite reductase), *norCB* (nitric oxide reductase), and *nosZ* (nitrous oxide reductase) (5). The aim of this study was to clarify the involvement of *B. japonicum* in the emission of N_2_O from the soybean rhizosphere. The N_2_O flux from denitrification mutants of *B. japonicum* was compared in the laboratory.

## Materials and Methods

### Bacterial strains, plasmids, and media

The bacterial strains and plasmids are listed in Table 1. *Bradyrhizobium japonicum* cells were grown at 30°C in HM salt medium (8) supplemented with 0.1% arabinose and 0.025% (w/v) yeast extract (Difco, Detroit, MI, USA). *Escherichia coli* cells used in transformation were grown at 37°C in Luria–Bertani medium (40). Antibiotics were added to the media at the following concentrations: for *B. japonicum*, 100 μg tetracycline (Tc) mL^−1^, 100 μg spectinomycin (Sp) mL^−1^, 100 μg streptomycin (Sm) mL^−1^, 100 μg kanamycin (Km) mL^−1^, and 100 μg polymyxin B mL^−1^; for *E. coli*, 50 μg Tc mL^−1^, 50 μg Sp mL^−1^, 50 μg Sm mL^−1^, 50 μg Km mL^−1^, and 50 μg ampicillin mL^−1^.

### Construction of *B. japonicum* mutants

**Fig. 1:**
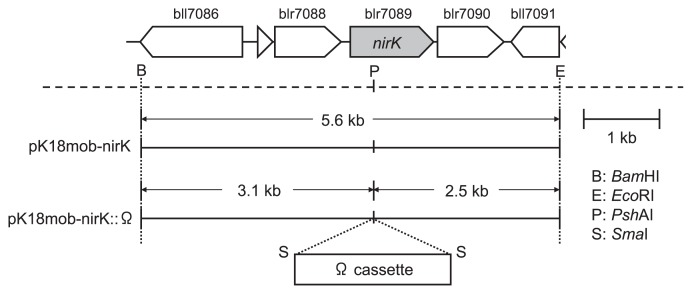
Construction of a *nirK* insertion mutant of *Bradyrhizobium japonicum* USDA110. Cloned fragments in pK18mob derivatives are shown alongside the physical map of the *nir* gene cluster of *B. japonicum* USDA110. See text for details.

Isolation of plasmids, DNA ligation, and transformation of *E. coli* were performed as described previously (40). DNA was prepared as described previously (43). A 5.6-kb *Bam*HI/*Eco*RI fragment containing *nirK* was isolated from brp01958, a clone from the pUC18 library of the sequences of *B. japonicum* USDA110, and inserted into the *Bam*HI/*Eco*RI site of pK18mob (Fig. 1). The Ω-cassette was isolated from pHP45Ω at the *Sma*I site (37) and inserted into pK18mob-*nirK* at the *Psh*AI site, yielding pK18mob-*nirK*::Ω (Fig. 1). pK18mob-*nirK*::Ω was introduced into *B. japonicum* USDA110 by triparental mating using pRK2013 as a helper plasmid (44). A USDA110Δ*nirK*Δ*nosZ* double mutant was constructed by the introduction of pK18mob-*nirK*::Ω into USDA110Δ*nosZ*::Tc (18) (Table 1). Double-crossover events of *nirK* mutation were verified by PCR.

### Preparation of soil suspension

Soil was collected from an experimental field at Tohoku University (Kashimadai, Miyagi, Japan). This gray lowland soil had pH[H_2_O] 5.6, pH[KCl] 4.2, total C 1.37%, total N 0.132%, and Truog P 48 mg P_2_O_5_ kg^−1^. Fresh soil (10 g) was extracted twice with 30 mL distilled water to remove nitrate and nitrite. The suspension was shaken for 10 min in centrifuge tubes and then centrifuged at 5,555×*g* for 15 min (Himac CR20E; Hitachi, Tokyo, Japan). The pellet was resuspended in 30 mL distilled water.

### Inoculation and plant cultivation

**Fig. 2:**
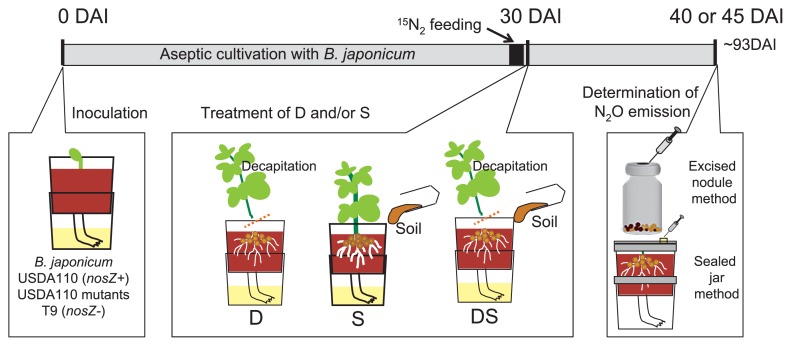
Soybean seedlings were inoculated with USDA110 (*nosZ*+), denitrification mutants of USDA110, or T9 (*nosZ*−). Plants were aseptically grown in Leonard jar pots (Fig. S2) for 30 days after inoculation (DAI). At 30 DAI, treatments were applied. At 40 or 45 DAI, gas phase was sampled for N_2_O analysis. In the long term monitoring, gas sampling was continued until 93 DAI. In the tracer experiment, ^15^N_2_ tracer gas was supplied for 8 h at 29 DAI. See text for details.

Surface-sterilized soybean seeds (*Glycine max* cv. Enrei) were germinated in sterile vermiculite for 2 days at 25°C. The seedling was then transplanted into a Leonard jar pot (one plant per pot) (28, 53, 56), which contained sterile vermiculite and nitrogen-free nutrient solution (31, 34) (Fig. S1). The seedlings were then inoculated with *B. japonicum* cells at 1×10^7^ cells per seedling. Plants were grown in a phytotron (Koito Industries, Tokyo, Japan) providing 270 μmol photons m^−2^ s^−1^ of photosynthetically active radiation (PAR, 400–700 nm) for 30 days at 25/20°C with a 16-h light/8-h dark cycle. A nitrogen-free sterilized nutrient solution (34) was periodically supplied to the pots. Thirty days after inoculation, a soil suspension (10 g in 30 mL) was added to the vermiculite in the pot (soil addition, S), or the aboveground parts of plants were excised (decapitation, D), or both treatments were performed (DS) (Fig. 2). The aim of the S treatment was to introduce soil microbes into the aseptic pot. That of the D treatment was to stop photosynthate supply to the soybean roots; because field N_2_O emission occurred more than 100 days after sowing (55), shoot decapitation was used to promote nodule senescence and degradation. The pots were left in the phytotron until N_2_O determination for 15 days except otherwise indicated.

### N_2_O determination

N_2_O flux was determined with a gas chromatograph (GC-14BpsE; Shimadzu, Kyoto, Japan) equipped with a ^63^Ni electron capture detector and tandem columns packed with Porapak Q (80/100 mesh; 3.0 mm×1.0 m and 3.0 mm×2.0 m).

### Model system for N_2_O emission from degraded nodules

USDA110 (*nosZ*+), USDA110Δ*nosZ* (*nosZ*−), and T9 (*nosZ*−) were used as inoculants. Thirty days after inoculation, treatments were applied (Fig. 2). Ten days later, nodules were collected from soybean roots, washed with sterilized water, and weighed. The nodules were introduced into a 19-mL airtight vial. Gas in the vial was sampled 1, 2, and 3 h after the vials were sealed to determine the N_2_O concentration. This was the “excised nodule method” (Fig. 2).

### Long-term N_2_O monitoring

T9 was used as inoculum. Thirty days after inoculation, the D or DS treatment was applied. The N_2_O flux from the pot was intermittently monitored during 2 months. On each measurement day, the pot was sealed with a lid with a gas sampling port (Fig. 2). After 5 h, the gas was sampled to determine N_2_O concentration. After the gas sampling, the pot was returned to the phytotron. This was the “sealed jar method” (Fig. 2).

### ^15^N_2_ feeding and ^15^N determination

At 29 days after inoculation, a gas mixture (30% [v/v] ^15^N_2_, 20% O_2_, 50% Ar; SI Sciences, Tokyo, Japan) containing 32.2 atom% ^15^N_2_ was supplied to the root zone of soybeans inoculated with USDA110Δ*nosZ* in seven pots for 8 h (Fig. 2 and S1). The nodules from three plants were separately collected and dried at 80°C for 3 days. The ^15^N concentrations of the powdered nodules were determined by mass spectrometer (EA 1110 DeltaPlus Advantage ConFlo III; Thermo Fisher Scientific, Bremen, Germany). The other four pots received the DS treatment. Fifteen days later, the gas phase was sampled by the sealed jar method (Fig. 2). The ^15^N concentrations were determined by gas chromatography/mass spectrometry (GC/MS-QP2010 Plus; Shimadzu) (21, 22).

### N_2_O emission from degraded nodules with denitrification mutants

USDA110, USDA110Δ*nosZ*, USDA110Δ*napA*Δ*nosZ*, and USDA110Δ*nirK*Δ*nosZ* were used as inoculants. Thirty days after inoculation, D or DS treatment was applied (Fig. 2). Fifteen days later, the N_2_O flux from the nodules was determined by the excised nodule method.

### N_2_O flux from soybean rhizosphere with denitrification mutants

USDA110 and its Δ*nosZ*, Δ*nirK*, and Δ*nirK*Δ*nosZ* mutants were used as inoculants to evaluate the effect of the *nirK* and *nosZ* genes on N_2_O emission from the rhizosphere. The *nirK* mutation was selected as a nitrate-to-N_2_O denitrification mutation, because the *nirK* mutant is not able to denitrify both nitrate and nitrite that exist in the rhizosphere (4). Thirty days after inoculation, DS treatment was applied. Fifteen days later, the N_2_O flux from each pot was determined by the sealed jar method, 3 h after the pot was sealed. In addition, 50 mL of 5 mM KNO_3_ solution was applied to each pot, the pots were immediately sealed, and the N_2_O flux was determined as above.

## Results

### N_2_O emission from degraded nodules

**Fig. 3:**
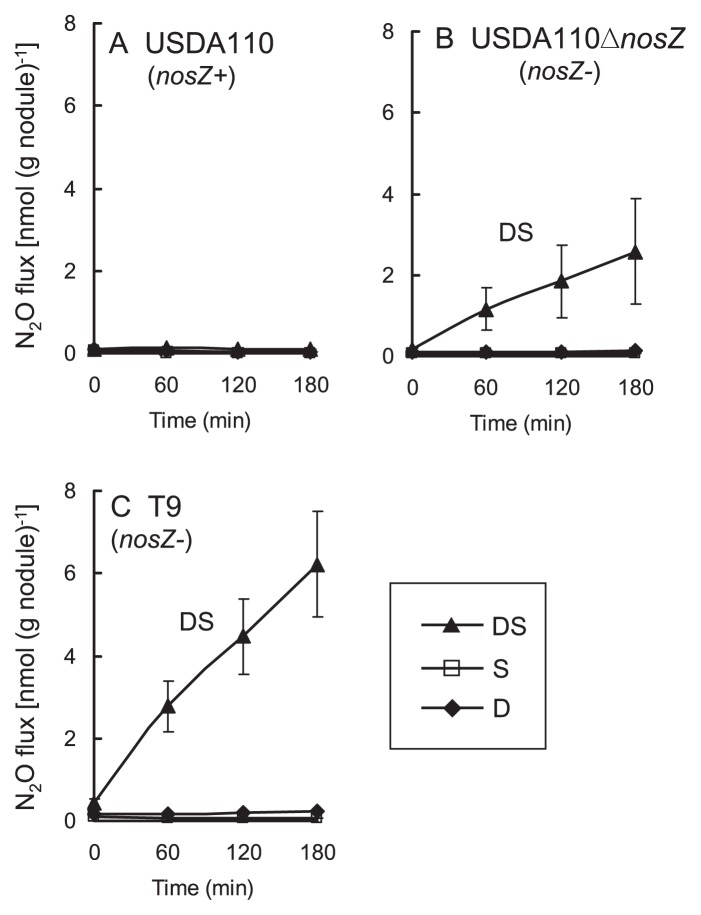
N_2_O flux (excised nodule method) from nodules of soybean inoculated with (A) USDA110, (B) USDA110Δ*nosZ*, or (C) T9 15 days after decapitation (D), soil addition (S), or both (DS). Bars indicate standard error with triplicate biological replications.

When *B. japonicum* USDA110 (*nosZ*+) was used as the inoculum, N_2_O was not emitted in any treatment (Fig. 3A). When USDA110Δ*nosZ* or T9 (each *nosZ*−) was used, the DS treatment induced marked N_2_O emission, whereas the D and S treatments alone did not induce N_2_O emission (Fig. 3B and C). Indeed, the nodules in the DS treatment were clearly degraded (Fig. S2), similar to those of field-grown soybean in the late growth period (20). On the other hand, the nodules in the S treatment stayed intact, and those in the D treatment looked slightly degraded (Fig. S2). These results indicate that both soil microbes and nodule degradation are required for N_2_O emission. In addition, N_2_O was emitted only from DS-treated nodules with *nosZ*− strains, suggesting that the *B. japonicum nosZ* gene is critical in the emission of N_2_O from degraded nodules.

### Long-term monitoring of N_2_O flux from the soybean rhizosphere

**Fig. 4:**
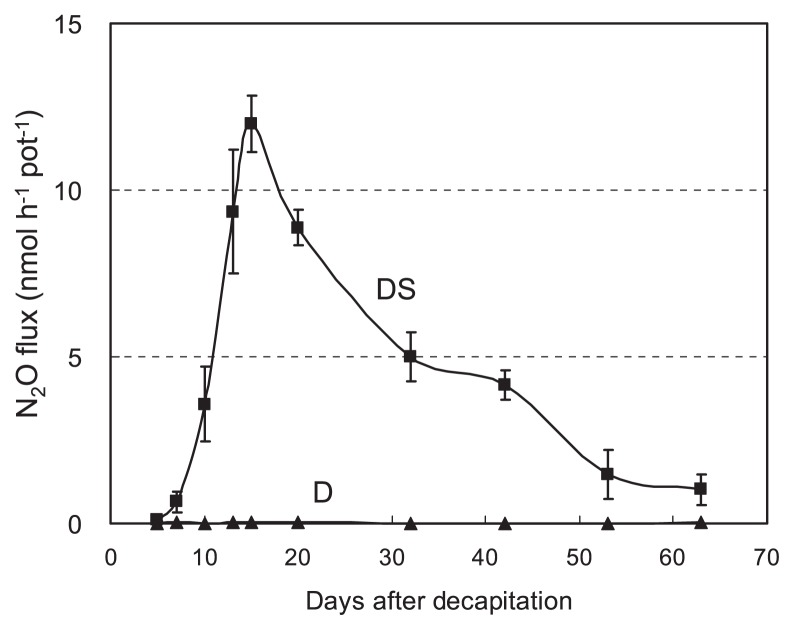
Long-term profile of N_2_O flux (sealed jar method) from the rhizosphere of soybean inoculated with T9 (*nosZ*−) after decapitation (D) or decapitation plus soil addition (DS). Bars indicate standard error with four biological replications.

Substantial N_2_O was emitted from the rhizosphere of soybeans inoculated with T9 (*nosZ*−) in DS treatment, but none was emitted in D treatment throughout the experimental period (5–63 days) (Fig. 4). This result is similar to the results in the excised nodule method (Fig. 3B and C). As the N_2_O flux in the DS treatment peaked 15 days after the treatment was applied and then gradually decreased (Fig. 4), we measured N_2_O flux at 15 days in later experiments.

### Origin of N_2_O-N

The profile of N_2_O flux (Fig. 4) suggests that the source of N_2_O was limited. Thus, we examined whether N_2_O is derived from N fixed in the nodules by using ^15^N-labeled dinitrogen. The supply of ^15^N_2_ to the root zone of USDA110Δ*nosZ* plants just before DS treatment produced ^15^N concentration in N_2_O emitted 15 days later of 1.32±0.42 atom% excess (mean ± SD), similar to the concentration of nodule N (1.13±0.08 atom% excess). This result clearly indicates that the N_2_O-N emitted from the soybean rhizosphere was derived from N fixed symbiotically in the nodules.

### N_2_O emission from degraded nodules with denitrification mutants

**Fig. 5:**
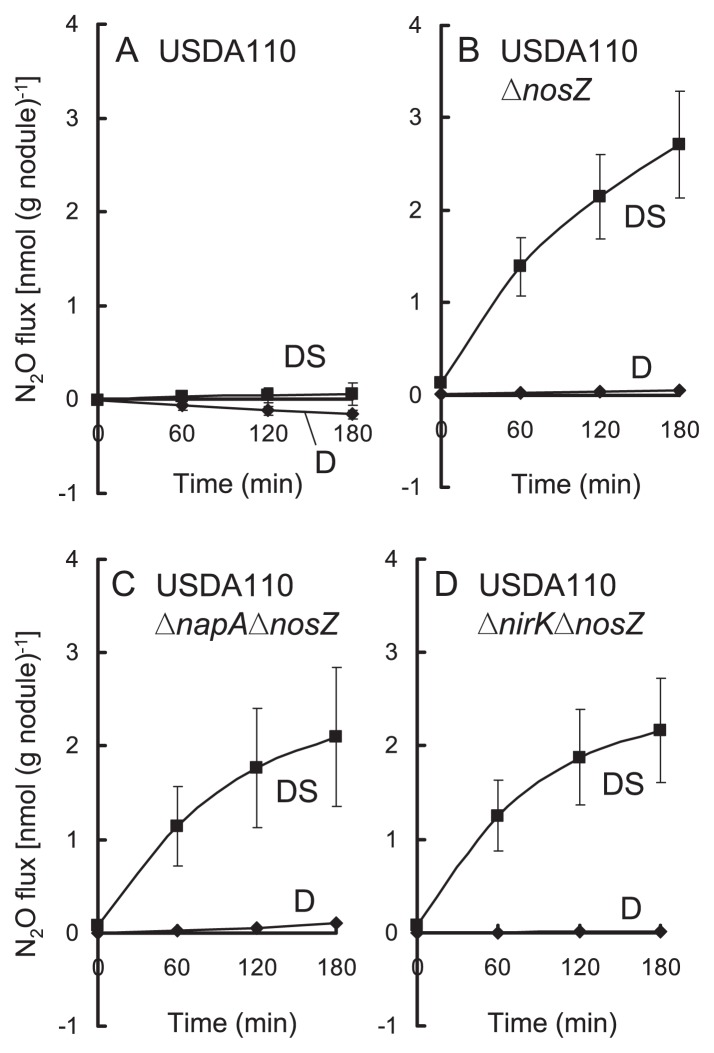
N_2_O flux (excised nodule method) from nodules of soybean inoculated with (A) USDA110, (B) Δ*nosZ*, (C) Δ*napA*Δ*nosZ*, or (D) Δ*nirK*Δ*nosZ* 15 days after decapitation (D) or decapitation + soil addition (DS). Bars indicate standard error with five biological replications.

N_2_O emissions from the nodules formed with USDA110 and its mutants were determined by the excised nodule method to reveal the involvement of bradyrhizobial denitrification (Fig. 5). Nodules inoculated with Δ*nosZ*, Δ*napA*Δ *nosZ*, and Δ*nirK*Δ*nosZ* emitted marked amounts of N_2_O in DS treatment. Nodules inoculated with USDA110 emitted negligible N_2_O even in DS treatment (Fig. 5A).

Because the *nosZ* gene is responsible for the reduction of N_2_O to N_2_ (18, 43), and no N_2_O was emitted from *nosZ*+ nodules (Figs. 3A and 5A), N_2_O reductase encoded by *nosZ* is likely a sink for N_2_O in the soybean rhizosphere. In the absence of *nosZ*, N_2_O emission from nodules inoculated with double mutants (Δ*napA*Δ*nosZ* and Δ*nirK*Δ*nosZ*) was lower than that from nodules with Δ*nosZ*, although there was no significant difference (Fig. 5B, C, and D, *t*-test [*P*<0.05]).

### N_2_O flux from the soybean rhizosphere with denitrification mutants

**Fig. 6:**
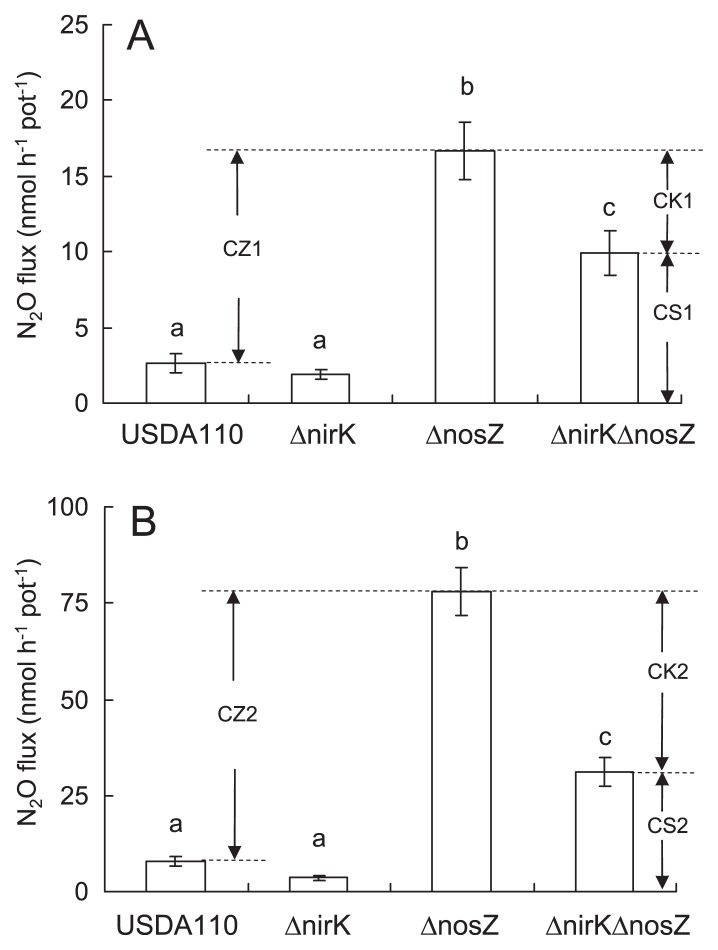
N_2_O emission (sealed jar method) from the rhizosphere of soybean inoculated with USDA110 or its denitrification mutants (Δ*nosZ*, Δ*nirK*, Δ*nirK*Δ*nosZ*) in (A) the absence and (B) the presence of KNO_3_. Bars indicate standard error with five biological replications. Differences in N_2_O flux are shown as follows: CZ1 and CZ2, contribution of *nosZ* in *B. japonicum*; CK1 (41%) and CK2 (60%), relative contribution of *nirK* under a Δ*nosZ* mutant background; CS1 (59%) and CS2 (40%), relative contribution of other soil organisms. Bars labeled with the same letter within a graph are not significantly different (*t*-test, *P*<0.05).

When soybean plants were inoculated with USDA110 and Δ*nirK*, a small quantity of N_2_O was released (1.9–2.6 nmol h^−1^ per pot; Fig. 6A). When plants were inoculated with Δ*nosZ* and Δ*nirK*Δ*nosZ*, N_2_O emission was significantly higher (16.7 and 9.9 nmol h^−1^ per pot, respectively). These results strongly suggest that the *nosZ* gene of *B. japonicum* is involved in the uptake of N_2_O that is released from degraded nodules. In Fig. 6A, the relative contribution of the *nosZ* gene to N_2_O flux is shown as “CZ1”. In the absence of *nosZ*, there was a significant difference in N_2_O flux between Δ*nosZ* and Δ*nirK*Δ*nosZ* (CK1 in Fig. 6A). This difference is due to the loss of nitrite reductase in the denitrifying pathway of *B. japonicum*. Therefore, the N_2_O flux from soybeans inoculated with Δ*nosZ* could have had two distinct sources; denitrification up to N_2_O by *B. japonicum* (CK1 [41%] in Fig. 6A), and other soil microbes (CS1 [59%] in Fig. 6A).

KNO_3_ was added to the rhizosphere to clarify whether NO_3_^−^ is a precursor of N_2_O. When KNO_3_ was supplied before N_2_O determination, the N_2_O flux from the pots with each inoculant was markedly enhanced, particularly from pots with Δ*nosZ* (78.1 nmol h^−1^ per pot) and Δ*nirK*Δ*nosZ* (31.3 nmol h^−1^ per pot; Fig. 6B). This result confirms that N_2_O was produced from NO_3_^−^ through microbial denitrification. KNO_3_ application also enhanced the contribution of *B. japonicum* to N_2_O flux (60% [CK2, Fig. 6B] cf. 41% [CK1, Fig. 6A]). These results suggest that *B. japonicum* prefers nitrate as a substrate for N_2_O production.

## Discussion

The term “rhizosphere” was first coined in 1904 by Lorenz Hiltner in Germany, who had a special interest in complicated N transformations around leguminous nodules with higher N contents in fields (16). In a sense, the present study advances such historical work on leguminous rhizospheres.

**Fig. 7:**
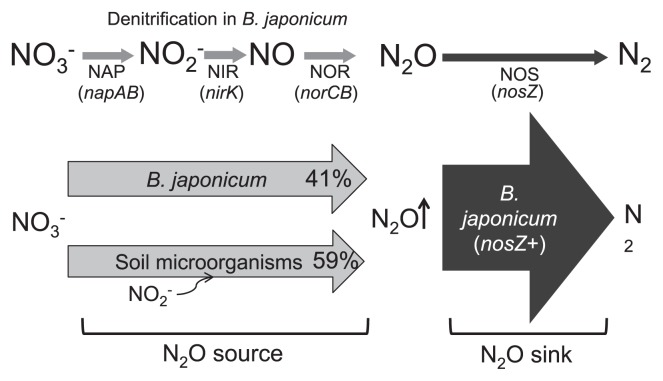
Schematic representation of N_2_O metabolism in the soybean rhizosphere induced from the present study. *Bradyrhizobium japonicum* and other soil microorganisms generate N_2_O during nodule degradation. *nosZ*+ strains of *B. japonicum* are exclusively able to take up N_2_O via N_2_O reductase. The relative contributions of N_2_O emission (CK1 and CS1 in Fig. 6 and text) are shown as percentages at arrows of *B. japonicum* and soil microorganisms. Net N_2_O flux is determined by the balance between source and sink. NAP, NO_3_^−^ reductase; NIR, NO_2_^−^ reductase; NOR, NO reductase; NOS, N_2_O reductase.

The results show that N_2_O emission from degraded nodules in the soybean rhizosphere is due to *B. japonicum* and other soil microbes. When plants were inoculated with *B. japonicum nosZ*− strains and treated with shoot decapitation and soil addition (DS), N_2_O was markedly produced (Figs. 3, 4, 5, and 6). On the other hand, when plants were inoculated with a *nosZ*+ strain, almost no N_2_O was emitted, even in DS treatment. These results suggest that N_2_O emission from degrading nodules formed with *nosZ*− strains was due to denitrification by both *B. japonicum* (*nosZ*−) and other soil microbes (Fig. 7). It is likely that N_2_O produced by soil microbes was offset by *nosZ*-competent *B. japonicum* with its N_2_O reductase. In other words, both *B. japonicum* and other soil microorganisms release N_2_O during nodule degradation (N_2_O source), and *nosZ*-competent *B. japonicum* (*nosZ*+ strains) takes up N_2_O (N_2_O sink) (Fig. 7).

What are these other soil microorganisms that emit N_2_O from degraded nodules? Prokaryotic denitrification, fungal denitrification, ammonium oxidation, and nitrate ammonification have been nominated as soil microbial sources of N_2_O (1, 14, 26, 38, 49, 50, 57). Community analysis specific to degrading nodules that emit N_2_O found many microorganisms that potentially produce N_2_O (20), including denitrifying bacteria such as *Acidovorax* (19) and *Enterobacter* (2); *Bradyrhizobium* (25), *Salmonella* (48), *Xanthomonas* (52), and *Pseudomonas* (36), which have functional genes and/or activities for denitrification; and *Fusarium*, a denitrifying fungus (45). Since *Fusarium* species generally lacks N_2_O reductase (51), it might be one of the key sources of N_2_O from degrading nodules.

The decline in N_2_O emission after the peak (Fig. 4) indicates that the source of N in the rhizosphere is limited. Indeed, the ^15^N tracer experiment showed that nodule N is a major source of N_2_O emission from the soybean rhizosphere. Thus, complicated N transformation in the soybean rhizosphere would involve ammonification, nitrification, and denitrification.

KNO_3_ addition enhanced N_2_O emission (Fig. 6), supporting the idea that NO_3_^−^ is a precursor of N_2_O. When NH_4_Cl was preliminarily added to the rhizosphere, the addition did not change N_2_O emission (Inaba *et al.*, unpublished data), suggesting that it is unlikely to be due to nitrification. KNO_3_ addition also enhanced the contribution of *B. japonicum* to N_2_O emission in relation to the other soil microbes (Fig. 6). Nitrate might be more available to *B. japonicum*, whereas other microbes might prefer other substrates. In fact, nitrite is a better substrate for denitrifying fungi to produce N_2_O (45). New approaches are needed to understand soil N_2_O-producing microorganisms and N transformation from fixed nitrogen in the rhizosphere (4).

In soybean fields, it is likely that soybean roots are infected with multiple strains that differ in denitrifying activity. *nosZ*− strains of *B. japonicum* that produce N_2_O as the denitrification end product often dominate in agricultural fields (3, 6, 11, 41, 42, 54). Both N_2_- and N_2_O-producing strains occurred in paddy–upland rotation fields (3). Similarly, both *nosZ*+ and *nosZ*− strains of *B. japonicum* were isolated from soybean fields (41, 42). Thus, it is easily conceivable that both N_2_-and N_2_O-producing strains of *B. japonicum* coexist in soybean fields. Consequently, the flux of N_2_O from soybean fields during the late growth period may be partly determined by biotic factors, namely the balance between N_2_O emission due to soil microbes and *B. japonicum* (*nosZ*−) and N_2_O uptake by *B. japonicum* (*nosZ*+) (Fig. 7).

The use of *nosZ*+ strains of *B. japonicum* as inoculants has been expected to reduce N_2_O emissions from soybean fields (42, 43). Indeed, *nosZ*+ strains produced no N_2_O and were able to take up N_2_O from degraded nodules (Fig. 7). Recently, N_2_O reduction by *nosZ*-carrying inoculants was shown in a soil-filled pot planted with soybeans (17). Thus, *B. japonicum* mutants with increased N_2_OR activity (23) might be more effective to reduce net N_2_O flux from soybean rhizosphere.

## Supplementary Material



## Figures and Tables

**Table 1:** Bacterial strains and plasmids used in this study

Strain or plasmid	Relevant characteristics^a^	Source or reference
Strains		
*Bradyrhizobium japonicum*		
USDA110	Wild type, *nosZ*+	25
USDA110Δ*nosZ*	USDA110 derivative, *nosZ*::del/ins Tc cassette; Tc^r^	18
USDA110Δ*napA*Δ*nosZ*	USDA110 derivative, *napA*:: Ω cassette, *nosZ*::del/ins Tc cassette; Sp^r^, Sm^r^, Tc^r^	18
USDA110Δ*nirK*	USDA110 derivative, *nirK*:: Ω cassette; Sp^r^, Sm^r^	This study
USDA110Δ*nirK*Δ*nosZ*	USDA110 derivative, *nirK*:: Ω cassette, *nosZ*::del/ins Tc cassette; Sp^r^, Sm^r^, Tc^r^	This study
T9	Field isolate in Tokachi, Hokkaido, Japan, *nosZ-*	42
*Escherichia coli*		
DH5a	*recA*; cloning strain	Toyobo

Plasmids		
brp01958	pUC18 carrying *nirK*	25
pHP45Ω	Plasmid carrying 2.1-kb Ω cassette; Sp^r^, Sm^r^, Ap^r^	37
pK18mob	Cloning vector; pMB1*ori* oriT; Km^r^	44
pK18mob-nirK	pK18mob carrying 5.6-kb nirK fragment; Km^r^	This study
pK18mob-nirK::Ω	pK18mob carrying *nirK*::Ω cassette; Km^r^, Sp^r^, Sm^r^	This study
pRK2013	ColE replicon carrying RK2 transfer genes; Km^r^	12

a Ap^r^, ampicillin resistant; Tc^r^, tetracycline resistant; Km^r^, kanamycin resistant; Sp^r^, streptomycin resistant; Sm^r^, spectinomycin resistant.
