# Supplementary material for: N2O Emission from Degraded Soybean Nodules Depends on Denitrification by Bradyrhizobium japonicum and Other Microbes in the Rhizosphere
Source: Microbes Environ. 2012 Oct 5;27(4):470–6. doi: 10.1264/jsme2.ME12100 (PMC4103556; doi:10.1264/jsme2.ME12100)
Supplement: Supplementary file 1 [file 27_470_s1.pdf]

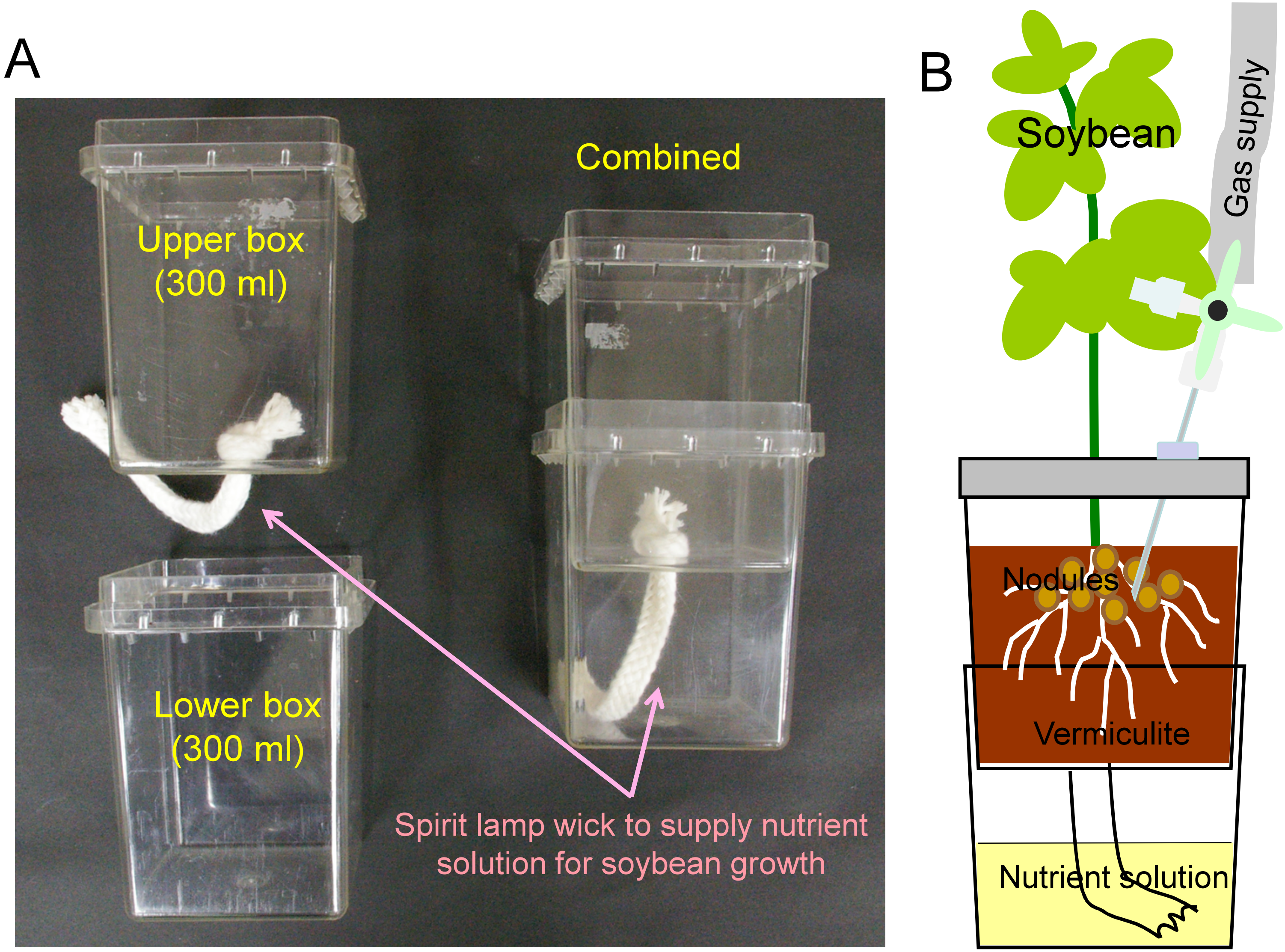

Fig. S1. Configuration of Leonard jar pots made of plant boxes (300 mL, CUL-JAR300; Iwaki, Tokyo, Japan). (A) The upper box with spirit lamp wick through a 7-mm hole in the bottom is inserted into the lower box. The upper box holds vermiculite. The lower box holds nutrient solution. (B) Delivery of $^{15}N_2$ to the soybean rhizosphere.

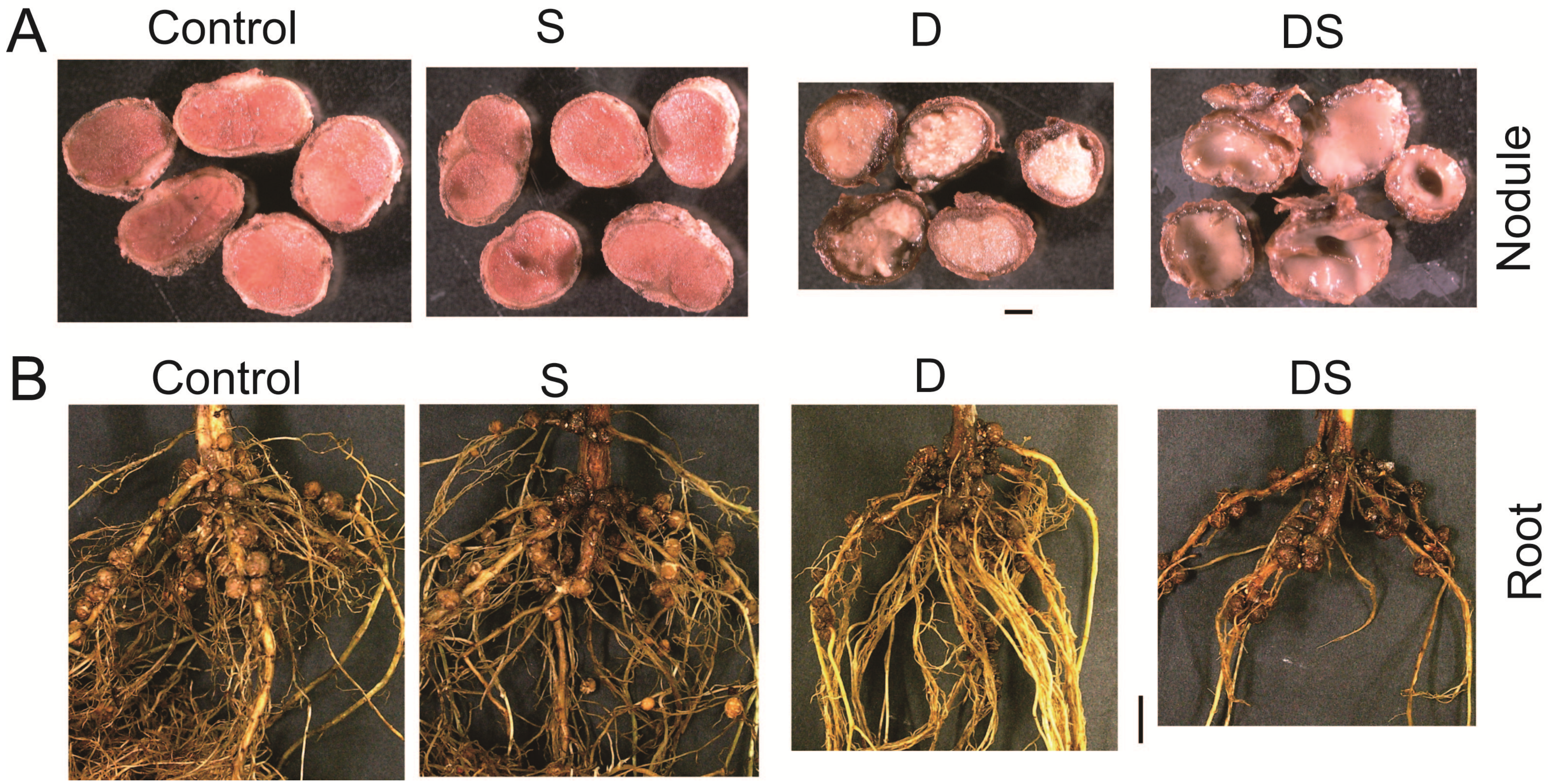

Fig. S2. (A) Nodules and (B) root systems of soybean inoculated with *Bradyrhizobium japonicum* USDA110Δ*nosZ* collected 15 days after soil addition (S), decapitation (D), both (DS), or neither (Control). Horizontal bar, 1 mm; vertical bar, 1 cm.
